# Identification of Novel O-Linked Glycosylated *Toxoplasma* Proteins by *Vicia villosa* Lectin Chromatography

**DOI:** 10.1371/journal.pone.0150561

**Published:** 2016-03-07

**Authors:** Kevin Wang, Eric D. Peng, Amy S. Huang, Dong Xia, Sarah J. Vermont, Gaelle Lentini, Maryse Lebrun, Jonathan M. Wastling, Peter J. Bradley

**Affiliations:** 1 Department of Microbiology, Immunology and Molecular Genetics, University of California Los Angeles, Los Angeles, California, 90095–1489, United States of America; 2 Institute of Infection and Global Health, University of Liverpool, Liverpool, United Kingdom; 3 UMR 5235 CNRS, Université de Montpellier 1 and 2, 34095, Montpellier, France; 4 Faculty of Natural Sciences, University of Keele, Staffordshire, United Kingdom; University of Georgia, UNITED STATES

## Abstract

*Toxoplasma gondii* maintains its intracellular life cycle using an extraordinary arsenal of parasite-specific organelles including the inner membrane complex (IMC), rhoptries, micronemes, and dense granules. While these unique compartments play critical roles in pathogenesis, many of their protein constituents have yet to be identified. We exploited the *Vicia villosa* lectin (VVL) to identify new glycosylated proteins that are present in these organelles. Purification of VVL-binding proteins by lectin affinity chromatography yielded a number of novel proteins that were subjected to further study, resulting in the identification of proteins from the dense granules, micronemes, rhoptries and IMC. We then chose to focus on three proteins identified by this approach, the SAG1 repeat containing protein SRS44, the rhoptry neck protein RON11 as well as a novel IMC protein we named IMC25. To assess function, we disrupted their genes by homologous recombination or CRISPR/Cas9. The knockouts were all successful, demonstrating that these proteins are not essential for invasion or intracellular survival. We also show that IMC25 undergoes substantial proteolytic processing that separates the C-terminal domain from the predicted glycosylation site. Together, we have demonstrated that lectin affinity chromatography is an efficient method of identifying new glycosylated parasite-specific proteins.

## Introduction

*Toxoplasma gondii* is an obligate intracellular parasite in the phylum Apicomplexa that is capable of infecting any mammal and causes serious disease in immunocompromised individuals and congenitally infected neonates [1, 2]. Other apicomplexans of medical importance in humans include *Plasmodium falciparum*, the causative agent of malaria and *Cryptosporidium spp*, which cause diarrhea in immunocompromised patients [3, 4]. The phylum Apicomplexa also contains a wide array of animal pathogens such as *Neospora caninum* (a pathogen of dogs and cattle), *Eimeria spp* (chickens), and *Theileria spp*. (cattle) that together cause substantial economic losses worldwide. Genome sequencing of these organisms has revealed that as much as half of their proteins are unique to these parasites and are not present in their mammalian hosts, thereby representing a large number of candidates for novel therapeutic interventions that target parasite-specific proteins and pathways.

The large number of parasite-specific proteins in the Apicomplexa is accompanied by a series of unique organelles that compartmentalize parasite functions. In particular, the secretory pathway contains specialized organelles named micronemes, rhoptries and dense granules that play critical roles in invasion, hijacking host functions, and nutrient acquisition. These organelles secrete their protein constituents at distinct stages during the invasion, resulting in the release of waves of secretory proteins to actively invade and hijack their mammalian host cells. Invasion begins when the micronemes secrete adhesins onto the plasma membrane of *Toxoplasma* to attach the parasite to the host membrane and activate gliding motility used for penetration via a parasite-derived actin-myosin motor [5]. After this initial attachment, the parasite reorients its apical end toward the host cell, followed by the secretion of rhoptry proteins [6]. The rhoptries carry two cargos from two distinct compartments of the organelle, the rhoptry neck (RONs) and the rhoptry body proteins (ROPs). A subset of the RONs is injected into the host membrane as complex that facilitates parasite entry. In contrast, the ROPs are secreted into the host cytosol where they localize to the parasitophorous vacuole (PV), the host nucleus, or remain in the host cytosol [7]. These ROPs serve as effectors that hijack host cell machinery to evade innate immunity and ensure the survival of the parasite within the host. Finally, the parasite secretes dense granule proteins (GRAs) into the PV to remodel the intracellular environment. Recently, a few GRAs have been shown to exit the vacuole into the host cytosol where they can translocate to the nucleus and modulate host functions [8]. Thus, *Toxoplasma* secretes micronemes, rhoptries, and dense granules in a temporal manner to ensure successful invasion and survival in the host cell.

Another important parasite-specific organelle is the inner membrane complex (IMC). The IMC is composed of Golgi-derived membrane stacks supported on a network of intermediate filaments. The membrane stacks are stitched together to span the parasite from the apical cap to the basal end of the parasite. The IMC plays a key role in host cell invasion by mounting the actin-myosin motor that functions in motility and host cell penetration, whose components together are known as the glideosome [9]. In addition, the *Toxoplasma* IMC plays a key role in endodyogeny, the unusual method of cellular division by internal budding in which two daughter parasites are formed inside of the mother and ultimately consume the mother to release the daughter cells. Disruptions of certain IMC proteins have resulted in endodyogeny or motility/invasion defects, highlighting the critical role of this compartment in apicomplexan infections [10].

Previous studies have shown that *Toxoplasma* has the capacity to post-translationally modify proteins via both N-linked and O-linked glycosylation [11, 12]. In *Toxoplasma*, the importance of N-linked glycosylation was revealed when parasites were treated with tunicamycin, resulting in replicative parasites within in the host cell, but which then become immotile and unable to invade new cells [13]. This defect was at least partially explained by the demonstration that the gliding associated protein GAP50 requires N-glycosylation for proper trafficking and association with glideosome partners [14]. Less is known regarding the role of O-linked glycosylation in apicomplexan parasites, which has mostly been studied by lectin staining in *Toxoplasma* bradyzoites [15]. Consistent with a primary role in encysted forms of the parasite disruption of TgNST1, which transports UDP-GalNAc, results in no apparent effects in the tachzyoite stage, but does affect the formation of bradyzoite tissue cysts in the brain [16]. Thus, the extent of O-linked glycosylation in tachyzoite and bradyzoite proteins in Toxoplasma and the functions of these proteins remain largely unknown.

In this manuscript, we have exploited the O-linked glycosylation system in *Toxoplasma* to identify novel proteins and examine their glycosylation and function in tachyzoites. We affinity purified a subset of these proteins using the *Vicia villosa* lectin that specifically stains *Toxoplasma* parasites and binds to O-linked N-acetylgalactosamine (GalNAc). Using this approach, we were able to identify known proteins as well as novel proteins that localize to the micronemes, rhoptries, parasitophorous vacuole and the IMC. We evaluated several of these proteins and assessed function via gene knockout. Together, this study identifies the major VVL-binding proteins in *Toxoplasma* and demonstrates that utilizing lectins to identify novel proteins is an efficient method to explore and screen for parasite-specific proteins.

## Results

### *Vicia villosa* lectin preferentially labels the *Toxoplasma* secretory compartments

**Fig 1:**
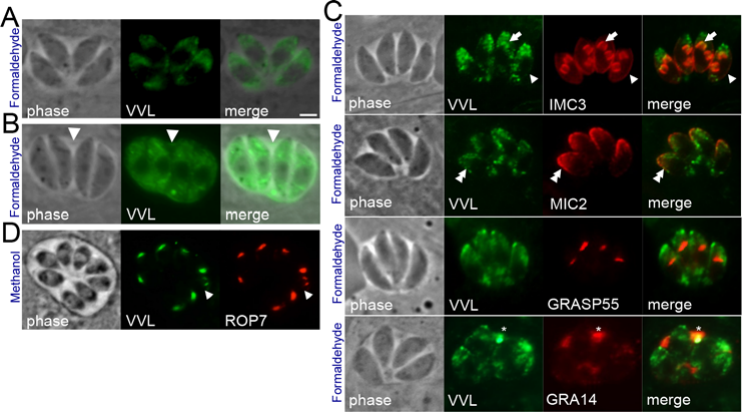
*Vicia villosa* lectin staining in *Toxoplasma*. (A-D) VVL-FITC was used to stain and visualize intracellular parasites using (A-C) formaldehyde fixation, or (D) methanol fixation. (A) Formaldehyde fixation results in both apical and posterior staining in the parasites, (B) some vacuoles also show staining in the vacuole (arrow). (C) Co-staining of VVL with IMC3 shows some co-localization at the periphery (arrowheads) and in daughter buds (arrow). Co-staining was also seen at the apical periphery with micronemes (MIC2, double arrowheads), and in the vacuole with GRA14 (asterisk), but not in the golgi (GRASP55). (D) Methanol fixation results in specific staining in the rhoptries as shown by co-localization with ROP7. Scale bar is 20μm.

To identify novel glycosylated proteins in *Toxoplasma*, we stained intracellular parasites with a panel of nine fluorescent lectins (Vector Labs, see materials and methods). One of these, *Vicia villosa* lectin (VVL), stained brightly in the parasites but not in the host cell. Surprisingly, the staining pattern with VVL-FITC varied dramatically depending on the fixation conditions. Using formaldehylde fixation, the lectin stains predominantly in the apical portion of the parasite with some posterior and vacuolar staining (Fig 1A and 1B). To evaluate what structures VVL is labeling, we co-stained the parasites with markers for various organelles, including the inner membrane complex (IMC3), the micronemes (MIC2), the golgi (GRASP55), and the dense granules/ parasitophorous vacuole (GRA14) (Fig 1C). We observed some co-localization at the periphery (Fig 1C, arrowhead) and in daughter buds (arrow) with IMC3. Co-localization was also seen in the apical end of the parasite with MIC2, and in the vacuole with GRA14, but not in the golgi. In methanol fixation, however, VVL stains exclusively club-shaped organelles in the apical end of the parasites, consistent with the rhoptries (Fig 1D). This was confirmed via co-localization with the rhoptry body protein ROP7, demonstrating that VVL does indeed label this compartment under methanol fixation conditions.

### Identification of VVL-binding proteins

**Fig 2:**
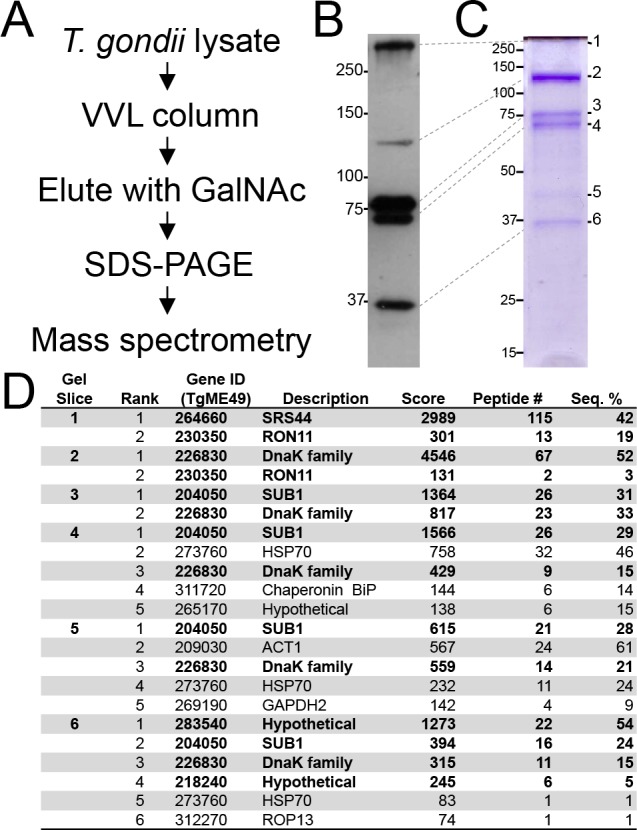
Identification of VVL-binding proteins. (A) Schematic for identifying VVL-binding proteins. (B, C) VVL Western blot (B) and commassie staining (C) of the eluted materials from VVL affinity chromatography. (D) Proteins identified from the VVL pull down. The bolded proteins are the ones we either discuss or study in the paper.

To identify the VVL-binding proteins in *T*. *gondii*, we prepared a large-scale lysate in radioimmunoprecipitation (RIPA) buffer and subjected it to VVL affinity chromatography. Following binding to the VVL column and washing, VVL-binding proteins were eluted using the competing sugar (GalNAc) (Fig 2A). We then ran the elution on a SDS-PAGE gel and detected VVL-binding proteins by Western blot and Commassie blue staining (Fig 2B and 2C). To identify specific proteins in each band, we excised the six most prominent bands, digested them with trypsin, and subjected them to mass spectrometry. The returned data provided us a list of the most abundant proteins that were eluted from the VVL affinity chromatography (Fig 2D, S1 Fig).

The most abundant protein in the eluted material was present in band 2 at ~125 kDa and corresponded with gene model TgME49_226830. This protein shares similarity with DnaK heat shock family proteins. BLAST analysis shows the highest similarity to a protein annotated as an ER lumen localized HSP70-like protein in *Neospora caninum*. Consistent with this, TgME49_226830 and its *Neospora* orthologue (NcLIV_046170) contain a predicted signal peptide at the N-terminus and the amino acid sequence REEL at the extreme C-terminus, a KDEL-like ER retention signal that has been documented previously in other ER proteins [17]. The next most abundant bands (bands 3,4,5) all gave top hits to the micronemal subtilisin-like protease TgSUB1, which has been previously shown to be processed into multiple fragments [18].

### SRS44 (CST1) is a vacuolar VVL-binding protein

**Fig 3:**
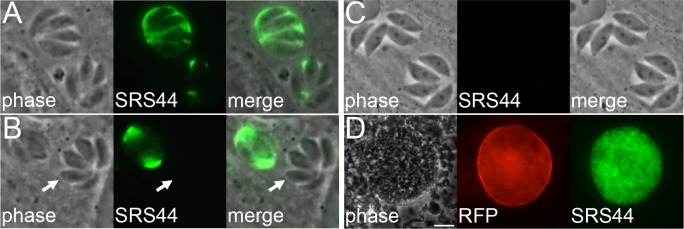
SRS44 is a vacuolar protein with 13 SAG repeats and a C-terminal mucin-like domain. (A-C) IFA labeling with SRS44 polyclonal antibody in either WT tachyzoites (A,B) or Δ*srs44* parasites (C). (A) Staining shows different levels of SRS44 expression between two different vacuoles (B) Staining shows SRS44 detection in some vacuoles but not all (arrow). (C) Disruption of *SRS44* was verified by IFA. (D) Mouse brain cysts of the pru*Δku80* strain have a GFP under a bradyzoites (cysts) promoter and show staining of SRS44 on the cyst wall. Scale bar is 20μm.

At the top of the gel of VVL-eluted proteins, a high molecular weight protein was identified which corresponds to SRS44, also known as CST1. SRS44 has recently been described as an important component for maintaining integrity of the bradyzoite cyst wall [19]. SRS44 is unusual in that it contains 13 tandem repeats of the SRS domain followed by a mucin-like domain and also lacks the predicted GPI anchor seen on most SRS proteins. Mucins are typically modified by O-linked glycosylation and are also known to bind VVL [20]. This region of SRS44 contains a stretch of over 100 threonines (interspersed with lysine, arginine and proline) that are highly predicted to be glycosylated by the NetOGlyc prediction program [21]. As current reagents only detect the glycoepitopes of SRS44, we expressed residues 803–1288 as a 6xHis tag fusion protein in *E*. *coli*, purified the recombinant protein using Ni-agarose, and injected the protein into mice for antibody production. Staining of intracellular tachyzoites demonstrated that SRS44 localizes to the parasitophorous vacuole. Surprisingly, SRS44 was detected strongly in some vacuoles, but was completely absent in others in standard infections (Fig 3A and 3B). The variability is seen in infections using both type I (RH) and type II (Pru) strain parasites and is more obvious in larger vacuoles (28 hrs, 4–16 parasites) than smaller ones (12 hrs, 2–4 parasites) (data not shown). To verify specificity of our antibody and assess the function of SRS44 in RH strain parasites, we disrupted its gene, which resulted in a complete loss of staining with the antibody (Fig 3C). As expected from its previous disruption in the lower virulence Pru strain parasites, we did not detect any gross defect of the knockout in culture of the parasites *in vitro*. The knockout parasites additionally retained their high virulence in mice, consistent with its role in the bradyzoite stage of the parasite (data not shown). Also consistent with its role in cyst integrity, SRS44 staining of bradyzoites from brain homogenates from mice infected with Pru strain parasites that specifically express GFP in the bradyzoite stage showed labeling within the cyst and bright staining of the cyst wall (Fig 3D).

### TgME49_283540 is a novel microneme protein

**Fig 4:**
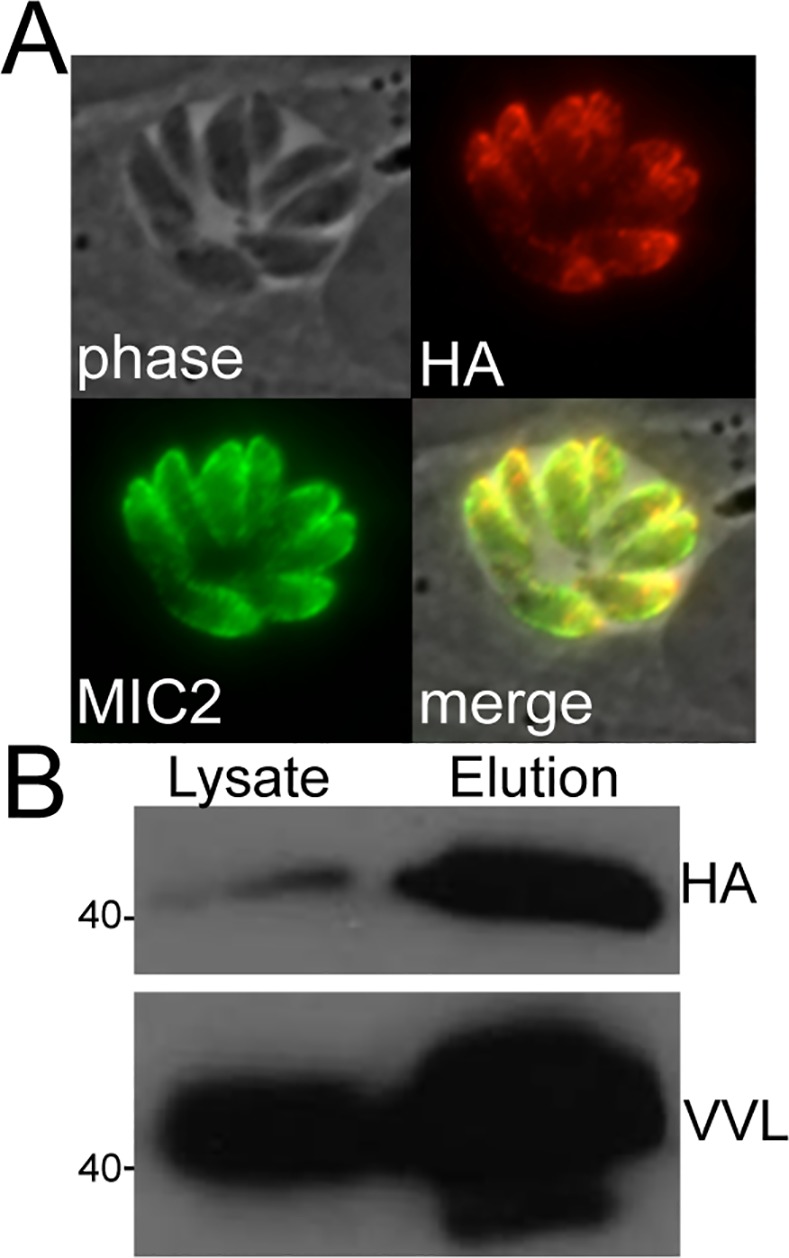
TgME49_283540 is a novel VVL-binding micronemal protein. (A) Heterologous expression of TgME49_283540-HA is detected in the micronemes, as shown by co-localization with MIC2. (B) VVL Western blotting of eluted materials from MIC20-HA pull down confirms that MIC20 is a VVL-binding protein.

The most abundant protein in band 6 corresponded to TgME49_283540, an uncharacterized protein that contains a predicted signal peptide suggesting localization to one of the parasites secretory organelles, but no identifiable homology to known proteins or domains that might allude to its function. To localize TgME49_283540, we amplified the entire coding region with the promoter and cloned this product into a vector that contains a downstream C-terminal HA tag and the GRA2 3’UTR. Expression of this construct in *Toxoplasma* revealed apical staining consistent with the micronemes. Co-staining of parasites expressing TgME49_283540-HA with MIC2 confirmed micronemal localization and thus we named this novel microneme protein MIC20 (Fig 4A). To evaluate whether or not MIC20 is likely to be glycosylated, we immunoprecipitated the protein with HA from *Toxoplasma* lysates expressing MIC20-HA. We obtained a strong enrichment of the target protein and stained Western blots of the eluted material with biotinylated VVL (Fig 4B). The VVL Western also showed strongly enriched staining of the eluted material, indicating that MIC20 is indeed O-glycosylated.

### RON11 is present in VVL elutions, and is not essential for growth *in vitro*

**Fig 5:**
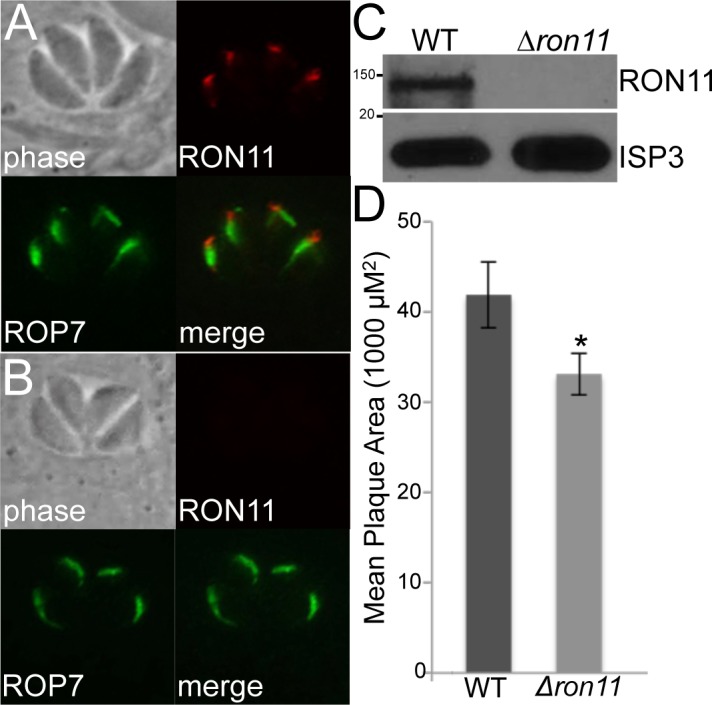
Δ*ron11* parasites show a minor fitness defect compared to the WT. (A) IFA demonstrating RON11 localization to the rhoptry necks in WT parasites, using previously described rat anti-serum raised against RON11[24]. (B, C) Δ*ron11* parasites were generated via CRISPR/Cas9-mediated disruption of *RON11* as shown by IFA (B) and Western blot (C). (D) Confluent HFF monolayers were infected with equal numbers of WT and Δ*ron11* tachyzoites. After seven days of incubation, parasites were fixed and stained with crystal violet as previously described [24]. For each experiment, 30 plaques were counted and measured from both the WT and Δ*ron11*. The bar graph represents the average mean for three experimental replicates. The error bars represent ±SD, and a paired two-tails t-test was performed comparing the mean plaque area of each Δ*ron11* experimental replicates to the WT to assess statistical significance (p = 0.033; *p<0.05).

Bands 1 and 2 also contained a rhoptry neck protein that we have recently identified and named RON11 (Fig 5A) [22]. RON11 was particularly interesting as it is conserved in apicomplexan parasites and also contains a calcium-binding domain in the C-terminal region of the protein. To assess function of this identified protein, we used a single crossover homologous recombination strategy to eliminate the calcium-binding domain. Integration just upstream of this domain eliminated staining with our antibody directed against the C-terminal calcium-binding domain. This integration event was intended to replace the C-terminal region with an HA tag but the tag could not be detected, suggesting that integration resulted in loss of the protein altogether, effectively generating a RON11 knockout (data not shown). A similar approach resulting in complete gene disruption was recently conducted with the micronemal protein AMA1 [23]. To confirm that RON11 is not essential, we disrupted its gene by CRISPR/Cas9 and confirmed the knockout by loss of staining with our antibody and Western blot analysis (Fig 5B and 5C). We first examined the Δr*on11* parasites for defects in invasion using 1 hour pulse invasion assays, but did not observe a significant difference in penetration efficiencies between wild-type and Δ*ron11* parasites. We also examined parasites over a longer time frame by plaque assays to detect more subtle differences in the lytic cycle (invasion, replication, egress) and did see a small difference in plaque sizes (Fig 5D). Our attempts to determine if RON11 is glycosylated using immunoprecipitations similar to that done for MIC20 failed to be conclusive (not shown).

### TgME_218240 is an IMC Protein that is Proteolytically Processed

TgME49_218240 also originated from Band 6 but was detected as a lower abundance hit by mass spectrometry (Fig 2). We suspected that TgME49_218240 could be a novel IMC or rhoptry protein because its cell cycle expression profile is similar to known IMC or rhoptry proteins, it contains a predicted N-terminal signal peptide for entrance into the secretory pathway, and it also has a predicted transmembrane domain in the C-terminal portion of the protein which could anchor it to the IMC or rhoptry membranes. BLAST analysis reveals that TgME49_218240 has orthologs in *Neospora* and *Eimeria* but lacks any identifiable domains and homology to any known proteins, suggesting that it plays a unique role in *T*. *gondii* and related parasites.

**Fig 6:**
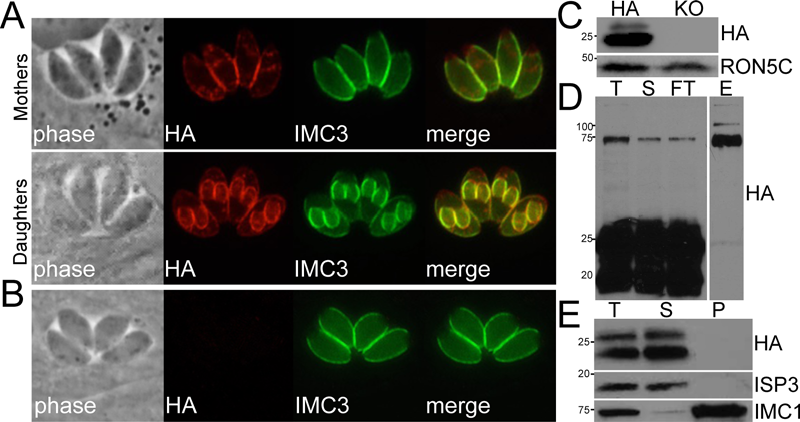
TgME49_218240 is an IMC membrane protein that is proteolytically processed. (A) TgME49_218240-HA shows IMC staining in both the maternal IMC and in daughter buds in parasites undergoing endodyogeny. (B, C) *The IMC25* gene was disrupted via CRISPR/Cas9 and Δ*imc25* parasites were confirmed by IFA (B) and Western blot (C). (D) VVL pull down Western blot probed with anti-HA antibody showing total (T), precolumn (S), flow through (FT) and elution (E). The eluted material detects the less abundant IMC25 precursors, indicating that the glycosylation is upstream of the IMC25 cleavage site (~20–25 kDa). This also suggests that the C-terminal fragments of IMC25 are not linked to the larger upstream glycosylated portion of IMC25. (E) Detergent extraction demonstrates that IMC25-HA is detergent-labile (T = total, S = detergent soluble, P = detergent insoluble IMC cytoskeleton), demonstrating that this portion of IMC25 is part of the membrane portion of the IMC. ISP3 and IMC1 are used as controls for the membrane and cytoskeletal portions of the IMC, respectively.

We then localized TgME49_218240 by endogenous gene tagging with a C-terminal hemagglutinin tag. Staining with anti-HA antibodies showed localization at the periphery except for a small gap at the extreme apical and basal ends of the parasite, characteristic of the IMC [25]. Staining of parasites that were undergoing endodyogeny showed that the daughter buds were also detected, demonstrating that TgME_218240 is indeed an IMC protein, which we thus named IMC25 (Fig 6A). To access the function of IMC25, we disrupted its gene using CRISPR/Cas9. A clone of *Δimc25* parasites was obtained by limiting dilution, confirming that *IMC25* is not essential. The knockout was confirmed by IFA and Western blot (Fig 6B and 6C). We examined growth by plaque assay and saw no gross defect in growth (data not shown). In addition, we did not see any defects in parasite endodyogeny by labeling daughter buds with ISP1 and IMC1 antibodies (data not shown)

Surprisingly, Western blot analysis of IMC25 on the epitope tagged strain revealed a predominant band at 25kDa and a less prominent 20kDa band whereas the predicted protein size is predicted to be ~132kD. This suggests that IMC25 is proteolytically processed, yielding 25 and 20kd C-terminal fragments. To determine if the C-terminal region of IMC25 is linked to the region of the protein that contains the sites predicted for O-linked glycosylation, we pulled down the glycosylated proteins with VVL (predicted to bind O-GalNAc sites on IMC25 upstream of the predicted cleavage event) and probed a Western blot of the lectin-pull down with anti-HA antibodies. Only the unprocessed (132kD) and partially processed (75kD) forms of IMC25 were present in the elution, indicating that the O-linked sites are upstream of the cleavage site. This agrees with O-linked glycosylation prediction programs that strongly predict O-glycosylation of a series of threonine residues that are upstream of the C-terminal region [21]. The result also indicates that the 25 and 20 kD fragments do not co-precipitate with the lectin-bound portion and thus these regions of the processed protein do not bind to each other tightly (Fig 6D). In addition, detergent extraction suggests that IMC25 is part of the membrane portion of the IMC as it is detected only in the supernatant with ISP3, a known membrane IMC protein (Fig 6E) [10].

## Discussion

In this study, we evaluated a panel of lectins for ones that stain *T*. *gondii* more strongly compared to host cells to provide an initial localization of glycosylated proteins as well as providing a straightforward method for isolating new proteins that might be involved in parasite pathogenesis. The most robust staining was obtained via VVL, which suggested targets in the vacuole, apical regions and rhoptries. The reason for enhanced staining of parasites is unknown, however, this lectin has a preference for single N-acetylgalactosamine residues and may require specific amino acid sequences at the site of glycosylation that could contribute to the staining observed [26].

VVL provides a useful tool for the identification of unique parasite proteins within the parasite whose glycosylation either plays a minor role in their function or can be compensated by other factors upon loss of function. The mass spectrometry from the VVL pull down resulted in the identification of six proteins from various subcellular compartments in *Toxoplasma*. The first of these hits, TgME49_226830, is predicted to be an ER localized heat shock protein that we decided to not pursue further. The second most abundant protein isolated protein is the micronemal processing protease TgSUB1, which processes adhesive complexes that are necessary for proper gliding motility, attachment, and virulence [18]. TgSUB1 has been previously shown to be processed into multiple forms, in agreement with the multiple bands isolated in our pulldown. Interestingly, previous work on SUB1 noted aberrant migration of the processed protein fragments, which is likely to be explained by glycosylation as suggested by its binding to VVL.

The next protein from the VVL pull down was the previously described bradyzoite cyst wall component SRS44 (or CST1), which was originally identified by dolichos biflorus agglutinin (DBA) lectin that also recognizes O-linked GalNAc [19]. SRS44 is an unusual protein with 13 repeats of SAG1-like sequences, no GPI anchor, and a C-terminal mucin-like domain that is strongly predicted to be O-glycosylated (similar to other mucin domains). While SRS44 is important for integrity of cysts *in vivo*, we see that it is also substantially expressed in tachyzoites. This is consistent with mass spectrometry and expression evidence from the *Toxoplasma* genome (www.Toxodb.org). The variability of SRS44 staining of individual vacuoles is surprising, and far more dramatic than any variability we have seen with other vacuolar GRA proteins. It is formally possible that these vacuoles are undergoing some stress and that staining represents an early transition to bradyzoites, but this seems unlikely as staining is present in the majority of vacuoles and we don't observe staining of bradyzoite markers (e.g. SRS9) in these samples (not shown). Alternatively, it is possible that SRS44 could be degraded in the environment of some vacuoles or that parasites within some vacuoles may cease to secrete the factor.

We also identified a new microneme protein that we named MIC20 via VVL affinity chromatography. MIC20 lacks apparent adhesive or transmembrane domains commonly seen in other microneme proteins. MIC20 could be a member of a multiprotein adhesive complex, similar to MIC2/MIC2AP, MIC1/4/6, or MIC3/8 [27, 28]. We did not address the function of MIC20 as BLAST analysis revealed that there is a paralogue for this protein (TgME49_239430) that also contains a predicted signal peptide in the *Toxoplasma* genome. Determining the localization of this protein will help to assess if it is a related microneme protein that may have a redundant or similar function to MIC20.

The fourth protein, TgME49_230350, has been previously identified by our lab as a rhoptry neck protein (RON11) that contains a calcium-binding domain [22]. Elevated cytosolic calcium released from intracellular stores has been shown to regulate the secretion of the micronemes, resulting in driving motility and invasion [29]. In addition, the subsequent drop in calcium is correlated with rhoptry discharge [30]. RON11 is highly conserved in apicomplexans, suggesting an important role in rhoptry-mediated functions. Surprisingly, we were able to disrupt RON11 without any gross effects on invasion or intracellular survival. It is possible that some other protein is compensating upon the loss of RON11, as compensation has been increasingly detected in knockouts in *T*. *gondii* [23, 24, 31]. BLAST analysis reveals one protein with low similarity to RON11 (TgME49_239430), but this gene is likely to be very lowly expressed. We attempted to endogenously epitope tag this gene in both wild-type and knockout parasites and could not detect any expression. It is also possible that RON11’s function is restricted to a particular host cell or host, which would be better evaluated by lower virulence strain parasites. Many RON proteins exist in protein complexes, thus identifying partners by pull-down experiments may help to reveal the function of RON11. To identify possible interactors, we attempted to use the *in vivo* biotinylation (BioID) approach that we recently adapted to *T*. *gondii*, but the fusion protein mistargeted and was inactive (not shown).

The last protein that was identified from the pull down is a novel uncharacterized protein, TgME49_218240 that localizes to the inner membrane complex of *Toxoplasma*, named IMC25. Western blot analysis suggests IMC25 is processed from 132kD to 25 kD or 20kD fragments. This is interesting because little is known about IMC protein processing events and this protein could provide information on the types of cleavage events that are present in the organelle. We presume that IMC25 is anchored in the membrane portion of the IMC via its transmembrane domain and is not tightly anchored to the cytoskeleton, as it readily released in TX-100 extractions of the parasite (Fig 5C).

Together, the VVL affinity approach provides an effective method for identifying novel parasite factors that are modified by O-linked glycosylation. While we have identified a number of the most prominent VVL-binding proteins, there are likely to be substantially more proteins that are lower in abundance and/or have few GalNAcs. The bulk of these would best be identified by deeper mass spectrometric analyses using MudPIT of the entire eluted fraction. In addition, this technique could be extended to determine specific sites of glycosylation by more in depth mass spectrometry of identified hits. While transport of GalNAcs via TgNST1 is not essential in tachyzoites, it is likely that these modifications affect the stability or activity proteins that are glycosylated. The development of methods for rapid endogenous tagging of genes in *Toxoplasma* combined with CRISPR/Cas9 based knockout systems make analyses of large numbers of these proteins even more feasible in the future.

## Materials and Methods

### Ethics statement

All animal studies were approved by the animal research committee at UCLA (ARC #2004–016, 2012–038, and 2012–077). The UCLA animal facility is accredited by the AALAC. All procedures including housing and welfare were carried out in accordance with the recommendations in the Guide for the Care and Use of Laboratory Animals of the National Institutes of Health. Specific details of our protocol were approved by the UCLA Chancellor’s Animal Research Committee (ARC#2004–055).

### Toxoplasma and host cell culture

*T*. *gondii* RHΔhxgprt (parental) strain and modified strains were maintained in confluent monolayers of human foreskin fibroblast (HFF) host cells as previously described [32].

### Antibodies

The following primary antibodies were used in IFA or Western blot: anti-MIC2 [28], anti-GRA14 [33], anti-ROP7 (Monoclonal antibody [MAb] 1B10) [33], anti-SRS44, anti-IMC3 [34], mouse anti-ISP3 [10], anti-IMC1 mAb 45.15 [35], rat anti-RON11 [24], VVL-FITC (Vector laboratories), and biotinylated-VVL (Vector laboratories). Hemagglutinin (HA) epitope was detected with mAb HA.11 (Covance) and rabbit polyclonal anti-HA (Invitrogen). For localization of GRASP55, a fluorescent fusion was used as previously described [36].

### IFA and Western blotting

For IFA, HFFs were grown to confluence on coverslips and infected with *T*. *gondii* parasites. After 18 to 36 h, the coverslips were fixed and processed for indirect immunofluorescence as previously described [37]. Primary antibodies were detected by species-specific secondary antibodies conjugated to Alexa 594 or 488. For the lectin staining, samples were incubated with a panel fluorescein labeled lectins diluted 1:300 (Vector Laboratories: *Vicia villosa* agglutinin, *Solanum tuberosum* lectin, *Lycopersicon esculentum* lectin, *Erythrina cristagalli* lectin, *Datura stramonium* lectin, *Griffonia simplicifolia* lectin II, Jacalin, *Ricinus communis* agglutinin I, Wheat Germ agglutinin). The coverslips were mounted in Vectashield (Vector Labs) and viewed with an Axio Imager.Z1 fluorescence microscope (Zeiss) as previously described [10].

For Western blotting, parasites were lysed in Laemmli sample buffer (50 mM Tris-HCl [pH 6.8], 10% glycerol, 2% SDS, 1% 2-mercaptoethanol, 0.1% bromophenol blue), and lysates were resolved by SDS-PAGE and transferred onto nitrocellulose membranes. Blots were probed with the indicated primary antibodies followed by secondary antibodies conjugated to horseradish peroxidase (HRP) or VVL conjugated to biotin followed by streptavidin-HRP. An ECL detection kit was used for the detection of HRP activity (Thermo Scientific) and target proteins were visualized by chemiluminescence.

### Affinity Purification of VVL-binding proteins

For purification of VVL-binding proteins, parasite lysates were made in 0.5% NP-40 buffer (50mM Tris ph 7.5, 150mM NaCl, and 0.5% NP-40) with Complete Protease Inhibitor Cocktail (Roche). Extracellular parasites (2 x 10^9^) were centrifuged at 3000g for 20 min. The parasites were washed once in 1X PBS and then lysed on ice for 20 min prior to removing insoluble material by centrifugation at 10,000g for 15 min. The vvl-conjugated beads were incubated with the lysate at 25°C for 2 hours before five washes with the lysis buffer. The bound proteins were eluted using lysis buffer supplemented with 100mM N-acetylgalactosamine. Five fractions were collected and analyzed by Western blot using biotinylated VVL (1:300).

### Generation of Polyclonal Anti-sera against SRS44

Sequences encoding amino acids 803–1288 of SRS44 were PCR amplified from a template of *T*. *gondii* strain RH genomic DNA with primers P1 and P2 (See Table 1) and then subcloned into the pET151GW-D-TOPO vector which encodes a C-terminal 6xHis tag for purification. The plasmid was transformed into *E*. *coli* BL21 (DE3) cells and grown to an *A*_600_ of 0.6 before the bacteria were induced using isopropyl-1-thio-d-galactopyranoside for 5 hours. Recombinant SRS44_803-1288_ protein with the 6xHis tag was purified using Ni-nitrilotriacetic acid-agarose chromatography under denaturing conditions and eluted using a low pH according to the manufacturer's guidelines (Qiagen). The purified protein was dialyzed against phosphate-buffered saline (PBS) and ~100 μg of protein was injected per immunization into BALB/c mice (Charles River) on a 21-day immunization schedule. The resulting mouse polyclonal antiserum was collected and tested by Western blot analysis and IFA.

**Table 1:** Oligonucleotide primers used in this study.

Name	Description	Sequence
P1	SRS44_803-1288_ for antibody production Sense	CACCAACTCTATGACGAGGCTGTCC
P2	SRS44_803-1288_ for antibody production Antisense	CCACATGAACAAGCAGGGACA
P3	SRS44 disruption 5’ Sense w/ *NotI*	GCGGCCGCGCTACTGTCGGTTGTTCCGGTTT
P4	SRS44 disruption 5’ Antisense w/ *SpeI*	ACTAGTGCCTCAGATCTCGGGGTTCTT
P5	SRS44 disruption 3’ Sense w/ *XhoI*	CTCGAGCCGCCGCTACAGTTTGTGG
P6	SRS44 disruption 3’ Antisense w/ *KpnI*	GGTACCGCCAACTGTTCACCCTCTCAAGA
P7	MIC20-HA Sense w/*KpnI*	CAGCGGTACCCCTTTTGTTCA
P8	MIC20-HA Antisense w/*NotI*	GCGGCCGCTATATTGTCATCTTGCTCGCCAGGACC
P9	RON11 gRNA Sense w/ *BsaI*	AAGTTGCGCTTCACGAACAGGGGAGG
P10	RON11 gRNA Antisense w/ *BsaI*	AAAACCTCCCCTGTTCGTGAAGCGCA
P11	IMC25 gRNA Sense *BsaI*	TACTTCCAATCCAATTTAGCCACGACCACGAAGACTCCCGCG
P12	IMC25 gRNA Antisense w/ *BsaI*	TCCTCCACTTCCAATTTTAGCGGCTGACTGTTCGATCAGTGGTCG
P13	IMC25 pHA-LIC Sense	TACTTCCAATCCAATTTAGCCACGACCACGAAGACTCCCGCG
P14	IMC25 pHA-LIC Antisense	TCCTCCACTTCCAATTTTAGCGGCTGACTGTTCGATCAGTGGTCG

To examine staining of SRS44 in bradyzoites, C57Bl/6 mice were infected with 500 Pru strain parasites that also contain GFP driven from the bradyzoites specific BSR4 promoter. At 31 days post infection, the mice were sacrificed, brains homogenized, fixed, and stained with anti-SRS44 antibodies and viewed for the presence of the protein and GFP by fluorescence microscopy.

### Disruption of *SRS44* via double homologous recombination

Deletion of the *SRS44* gene was accomplished by homologous recombination using a construct derived from the pMini-GFP.ht knockout vector which contains the selectable marker hypoxanthine-xanthine-guanine phosphoribosyltransferase (*HPT*) and also contains the green fluorescent protein (*GFP*) as a downstream marker to distinguish homologous and heterologous recombinants [38]. The 5′ flank (1,320 bp) and 3′ flank (1,256 bp) of *SRS44* were amplified using primer pairs P3/P4 and P5/P6, respectively. These genomic flanks were then cloned into pMini-GFP.ht upstream and downstream of *HPT*, resulting in the vector pSRS44-KO-HPT.

30 μg of pSRS44-KO-HPT was transfected into RHΔ*hpt* parasites and selection for *HPT* was applied 12 hours post-transfection using 50 μg/ml mycophenolic acid and 50 μg/ml xanthine. Surviving parasites were cloned by limiting dilution eight days post-transfection and screened for GFP by fluorescence microscopy. GFP-negative clones were assessed for absence of SRS44 staining by IFA.

### Virulence studies with *Δsrs44* parasites

To evaluate the virulence of *Δsrs44* parasites, 100 knock out parasites were injected into female 6-week old C57Bl/6 mice. All mice became moribund with the same kinetics as infections with wild-type parasites, demonstrating no change in virulence *in vivo*. All procedures were in accordance with the UCLA ARC (protocol #55–044).

### Second copy-expression of TgME49_283540, MIC20-HA

To epitope tag TgME49_283540, the entire coding region and additional 2 kb sequence upstream of the gene was amplified using primers P7/P8 (See Table 1), respectively, and cloned into the pNOT-HA_HPT vector, which encodes a C-terminal HA-tag [33]. The constructs were linearized and transfected by electroporation into *T*. *gondii* strain RHΔ*hpt*. The transfected parasites were grown in medium containing 50 μg/ml mycophenolic acid (MPA) and 50 μg/ml xanthine, and selected parasites were cloned. To assess targeting, parasites were analyzed by immunofluorescence with rabbit polyclonal HA antibody. Immunoprecipitations of MIC20 using the HA tag were carried out as described [22].

### Gene disruption via the CRISPR/CAS9 system

The knockout of RON11 was accomplished using the CRISPR/CAS9-based system in *Toxoplasma* as previously described [39]. The 20bp protospacer used by the CAS9 system was designed to make a double stranded break in the *Toxoplasma* genome in the first exon of *RON11*. The protospacer was made by annealing primers P9/P10 (See Table 1) and cloned into pU6-universal vector via BsaI sites. The final vector was co-transfected into WT parasites with an *HPT* cassette at a 5:1 ratio respectively. Parasites were selected with 50 μg/ml mycophenolic acid and 50 μg/ml xanthine. Eight days post-transfection, surviving parasites were cloned by limiting dilution and screened by IFA for *RON11* negative parasites using the RON11 antibody. Disruption of *IMC25* was similarly conducted with primers P11/P12 (See Table 1).

### Plaque assays

Parasites were grown 48 hrs, syringe lysed and infected into 6-well dishes containing fresh, confluent HFF monolayers. Cultures were allowed to grow seven days for RON11 and nine days for IMC25 before fixation with methanol followed by staining with crystal violet as previously described [24].

### Epitope tagging of IMC25

For endogenous tagging of IMC25, we used the plasmid p3XHA-LIC-DHFR [22]. The 3’ portion of the *IMC25* was PCR amplified with P13/P14 (See Table 1) and inserted into the plasmid using a ligation-independent cloning approach (LIC) to generate a 3XHA epitope tag fusion prior to the stop codon of IMC25. The constructs were linearized within the amplified region and 50 μg of DNA were transfected into RHΔ*ku80*Δ*hpt* and cloned by limiting dilution after selection in drug medium containing 1 μM pyrimethamine. Clones that had undergone the intended recombination event were screened by IFA and Western blotting against the 3XHA tag with mouse monoclonal HA antibody used at 1:1000.

### Detergent extractions

Extracellular IMC25-HA parasites were washed in PBS, pelleted, and lysed in 1 ml of 1% Triton X-100 lysis buffer (50mM Tris-HCl [pH 7.4], 150 mM NacCl) supplemented with Complete protease inhibitor cocktail (Roche) for 30 min on ice. Lysates were centrifuged for 15 min at 14,000 X g. Equivalent amounts of total, supernatant, and pellet fractions were separated by SDS-PAGE and analyzed by Western blotting using ISP3 and IMC1 for controls as described [25].

### Accession numbers

Genbank accession numbers: KU550704 for IMC25 and KU550705 for MIC20

## Supporting Information

S1 Fig Peptides identified in VVL-binding proteins via mass spectrometry. The peptides that were identified from mass spectrometry in each VVL-binding proteins are highlighted in red. Some of the identified peptides were derived from multiple bands.
(PDF)
