# Supplementary material for: Identification of Novel O-Linked Glycosylated Toxoplasma Proteins by Vicia villosa Lectin Chromatography
Source: PLoS One. 2016 Mar 7;11(3):e0150561. doi: 10.1371/journal.pone.0150561 (PMC4780768; doi:10.1371/journal.pone.0150561)
Supplement: S1 Fig — The peptides that were identified from mass spectrometry in each VVL-binding proteins are highlighted in red. Some of the identified peptides were derived from multiple bands. (PDF) [file pone.0150561.s001.pdf]

**S1 Figure.** Peptides identified in VVL-binding proteins via mass spectrometry.

## SRS44

MTAPFLRVYTDILSERKVRVEGTWHLVSIKATHDQPLCACKQTWSVHFAWPVDTMKLSYFFFALSSSGATLLLFAVTQLATQVSGFDLEEPRDLRHEMK**KIEVIHG**
**SEGARFECPVESVLLPEQAMSGDIR**AATVFAVAADGSCNIFDPPSLLGDVVPGAYLQEELSVNADKAAK**TYTLVIPGLPAKEVKLCYVCAHPDNPAGSQVTVITVR**
**R**SGDSENGNPASSPVCRPSFSLEAWALGVR**QPSVVLDVAAPGQSVRFSCLSTATELPSEAGARPAPAQVYPVNGRGWCDLSKGPSSLEKFLLGAAMTAEVR**QR**GGG**
**FLTTYTLNVPR**LPSTNR**RFCYVCPRNRDPADGCAVLVKVRGGMVEDPIGKPAAVPTCHPQESPEGIAAGTKSAALILDIVRPYSSVKFACPNNSEVLPHNAVSDDG**
**LKASVFAVRKDGSCIIGEHTLKLQDVVPGATLTGTTLVDKGSVTRVYTFFVGMLPTR**SQKVCYVCAR**NSLAPQGCGVVISVK**GKENGKPAK**VSVCEPQYLQGVAER**
GLLSPSVELLVDKPEGVAQFVCPGNSVVFPEHATDESPGIATVYQASNANACDVWENPVK**LDNVLQGATLR**ADTSDEDTMMAKTYTFKVPALPSTETKLCYACVR**D**
**GEIANSCGVMITVKPKGPTTEEIPSALLERGLCLPVYSTDVAK**TGLRRPSVVLKVDKRNRVVEFTCPHSSLVFPDQAADSDAGEAVVYTVSGGSCDVWENPVYLTK
**ILPGASLVSETAVDASEITTK**YTFTVKQLPKTPK**KLCYLCVYNRDVMNSCGVLIDVSSNSMTRLSKTSEEGIHTNPTVPTTLELQCPPDYSARTAATGLRLPEMEL**
**LVDSVNR**TAR**FVCPDDSSVFPEIDGEADPASGKVYRMTGDSCDTREETAFLQDEVPGAELQRRTVVDGGVVQR**YYIFKASRLPEEDKK**LCFLCVRQRNPADSCAVM**
**ITVK**ADSAAAVPIHPFRPRPAVKLATYVCKPENSTEVAETGLK**APALSLTIDSR**AHSIQFICPESSIVFPDLAAVPDQDSATVYLVGEGGSCNVAASPR**LLSDVLP**
**GATLGSRTTVDEGVVNRTYTLTAPKLPEEDVKLCYLCVRNRQIAESCGIHITVKGTKAESVAKPAGPICSLGYSPGSEKTGMREAAIELVVEDPSTPVRFICPEGS**
**YVFPEYATSTDKGIAAVYGVKGNECIFAGPRENLQSLVPGATLRMEAADEGGTVMRTYTFTTPEQPAEDRRFCYMCVRERDIYQSCPVIIVVK**GSGHLPEPK**AQPQ**
**EEHELPVCTPHEQAGTALTGLLDPSVK**LWVNEPEEQVSFVCPDGSAVLPDQETSEPQVYAVGAGEYCLVTKPLMSLAK**AVPSASLSTDTVVENEVTTR**KYTLR**VPS**
**LPLEETKLCYTCVSEVDPAKKCGVSVTVK**GEGKMPGGLTSSLPSPVCAAQFSQCAFLTGVR**MPSIMLTVHEPNTKLVFDCPPNTALYPQNAVVPGKATFFPLNEKG**
**ACIYKGPVAESKLYDVVPGAR**LRAK**ETVDGAVVRSYTLTVSKLPK**KETR**LCYLCVRGDEPAYSCGVIVKVKAPAPAAPEPKQLPVCVPSSDPSVGK**MRRITPKLTL
K**IEKPGGSASFECPDGSTIFPDHADALDPK**CVLAYKVVR**DTCDLRGPTEMVASKLQGAYLTKTFVGTGYK**YTFTVPHLPAEQQQVCFMCVMDRDTSR**SCGVVINVM**
**PHPAASR**SSVPECKLK**HVPAAATTGLREASLILNVREPSSVVEFGCPRNSVIMPSTATSSEHLAQVYSVRDRHICDMKEFLRPLADLVPGASLKLVHTPEDGDTTR**
**THMFR**VGSLPVKDTALCYLCVDPGDHSHNCGVVINVK**GSEVPPPPKDFGCAPSHSEEVERTGK**LSPAVR**LMVTSTKEPIR**FECRDNAK**VYPPAASLIKPAKAVVYP**
**VSAGGRCIVGKPPLSLNR**VVPGAKLKTKAKLVTGAITTTGFVFVPVLPSIQK**DFCFACVDR**GDITNSCGVLVTVPPFWAPPVQETVCNDVKTLTMTVHSPGSVTLR
CGLEFPKVDPPIDDPASQGQVYYGKSCGDVGALRTVIPGASVVKEPFYHNLAKEVAVGVTLTVPASIVKQTICFKCRQPDPLQHETCTVRVTVLPPAAQPTTTTTT
TTTTTIKPTTTTTTRKPTTTTTTTTRRPTTTTTTTKKPTTTTTTTTRKPRTTTTTTTKKPTTTTTTTTRKPTTTTTTTKKPTTTTTTTKKPTTTTTTTKKPTTTTTTTT
TRKPTTTTTTTKKPTTTTTTTKKPTTTTTTTTKKPTTTTTTTKKPTTTTTTTTRKPTTTTTTTTRKPTTTTTTNKPTTTTTTTRTPTTTTSKPPVVFELPSEFIEETAT
TTRATISLRARSAAAPTSTTTVGPTTPATSLFDWSSKVASLAGQWSFSGALQGTAGSRSGVYEAPITQLPPPLLVPFKIDCCGPPVIQKGCCPPPLIQVDCCRPPV
LQQKPGCCCCGHE

## RON11

MGWVPPCAAPAHSQAAANLRVESLGSRSFPDGSTKKRRREERTDRFRNLHFRTLLYSCSFSCSRNAPANGHKTSSPGHPEESLRPTPLFVKRARFCFYLLFLFSVL
ASNFFSHRADETRFCNVFAQALHVKLDNPELSRQTLPASLRWQQVKTKNGEEVNAAESASWKGFADVQLAFRPDEKPSRERTVHEHRDTVHGLSGRDTLGIDSFRG
TDLRGNRLFVRLPRPALHSPFLFSFSFPAATQPAESSVFFGADGIPSFSEIHSEQSEDAEEPESQDSSSSRNQDEAPKPTEHSQEKAHESQGEPTEDSGAAESASE
HPSPSNSIPAEEESSATATQEKPTEGPSAPQTLASLGGDTASSSGTEQTERRSGSPVEEQGKDSTRTASLGSVSYSSTTTTTGTPSAFNVK**EALLAPVPTGAVAGP**
**LAGR**EARAQRGASADSGERGAAGAKEAAGEAQGPSEVSEEKAEPKQKETEGGKTGGDKDGQER**GSAAGKEDSAPTTEKPLSK**AEMERAAAEFVKKEGGMGSLEK**KL**
**AESGPQLTEEQTKTFVDTQEVAQNVR**KLIGLKAGSNLHLENQKLIK**ADCDLATFGPEYCAAR**DNPNALSLLWSLEHRK**NTIYFVIAMITLAFGIAVQTCIR**LLERK
ISEGRDEFSKEVMETFRQIATISIVNVILWGTMQSNVAEVLDELVFGDVMPPLRDSDTVLENVSPMLEVLFEELFVSVILLVWYVVFVILFQCMIRQIIEWMRKID
EEEDIPLIARDAETQSHRWCGAVLGRSLDKANFIAHRYEFTDNVQSMSIPGVDPSGYYFMEYLRASLLKMAIEMIRLPNSTLFFLMIMDICLRPTFSFRLGQEVGL
ISGLSATCLVGMLLVWAYTARIEKQLLPRHVPHYLVMRYHMEVGGDTSSEYLKEFAPPYKLQKQKNAWPGVCSTFLRGTTLPNKHQQLFCFWANGPNIVLRAVEAS
FFCQLLVLAWWVQLFRSHPATWLNFCWWGNVVVVLCAGSQFALLKSVVYTVLLALNSGMLVDPELLEKVWDLQRAENVRRTAELIDALR**VQSTLFAISEGGDMFWR**
QLLIRSKGASRQAQEQRLSLWAALDEEHQGEISRAKIFK**FLTSQGLPIKSESGVNDFLHVFDR**NHKGGVNEDEFFVMVMVVKQMLMEPLDRDAVRELFEGR**YGIPW**
**TSPVGIDLANLSK**ILAELRLTLSEGKKR**YLLDFIGGK**R**GTTAVSPDHFVSQLQAMEEQALNPIQSESLRGPPIPGERV**

## DnaK Family

MALPAFEGVSPCVGGHRRVSPSGSSVRSLSHSSRLGCFFFAFCFLSVSLFSPLGAKAQVVGLDLGSEFVK**VALVAAGRPIEILLNPASKR**K**TNNAVSFADEEKREL**
**GDEGAAQAAKKPDR**VFLHPNLLLGVNATDFGLVDVTADPDSAFLPLALK**EKLLPSGYPHDYYPYRLFMDR**RR**HSVAVLAKDGVYLPAELLTASMLAFVKKLATQAA**
**GVDNEKTLGCVISVPCR**YTQRQRQALRDAVEIAGMHAVAFHHHSVTAAVQHALDLPTNTTATK**LFYDVGSSTIDVGVVRFAPVQLPSKKEVLQVQLLACESMSGAG**
**GHHVDLAIAEK**MRGAFERRHGAGK**SLLGVPR**ALKKLVKQAVMAK**HVLSANKQTQFRVEGLHNDVDFHEPFERMHLEALLEEKGMLAKLKASLDATLSHAGLDLNDV**
**DQVELLGGASRVPRVQQELGALMGAK**DVGTHLNGDEAMATGAAFIAANSTATFR**VKQLLLQDITPYEYLVKIDAVAEEDDPDAGVR**RGDRVKKTKVLVGRHARFAQ
GSRTVSLRTTQDFQVELFEDDASLLK**LAVTGVAEAAKK**LEEEAQKKAEAESKTEKNGEVESETPERRLPK**ANLVFKVDGAGVLSFDR**AYLTQDYFVYVPLPSTTTK
ATTTK**ESEGEETSTTTPAPAAK**FKR**LTKQVSDALAVEETQTLPLPLTSEER**NALR**KQLQEMEAADLRVHKLAEAMNRLEASIYAARGLLAEETVERVSLPETREEL**
**RKQLEADEEWIYDSAKAEGIEAVK**K**KLVALESVVEPMKKRAEELEARPK**AIEKMEK**ALVDLVSRLEQIATKRPWVSEEK**LASVR**AFGQDVDAWWREKLASQQSKLD**
**TEDPVFTKAEVLAK**VKELGAAVKALENIPRPTTTTTTSPGEGKEPGEQKQEEKTEEKKQEEKNEEKKEEEKNEEKKEEKVEEEHKNREEL

## SUB1

MGSSHAIVACAALIVLLSTNARGLRVRKDKDVLLATSFLSHHGEYQNPTGTYNLIKEIRRVEAEIEDEVETLNRDRRLHRGHNKYADDDIRQGLKGEQDMGASENI
PVAELEPQDLDREAKYPVRMLIVDKRSDDDDEETKTSFVETALHSDLAQRVVKELNGHVDVLRESGVVLVDLPAQTTDKQLQELIETARAQGTIVEPDHLVQSVNT
SSK**GSNDPLLDRLWGMDALNVK**EAWDIITTGEPNMGGR**RPLVCVLDTGIDYNHPDLRDNMEVNQAER**DGTLGVDDDNNGEVDDIYGANMLSKENDPADDHSHGTHV
AGTIGAHGNNGIGVAGVAWAPR**LLPCKFLAYTGRGYSSDAVRCIDYCVKRGADIVNHSWGGSWPSEALREAVVR**TANNGLIHIFAAGNDGVDIDQTAFYPAAFSTE
ADGLITVANVKGDPDHGGK**RIIELDRSSNYGIQRVQVACPGMWILSTVPTSGSSQEPYAEKSGTSMAAPALSGIVALMLAVNPGLSTR**QVR**EGLRQCSVQQPLLQG**
**KVEWGSMPDAKRCVEYALTTHAEGRHK**SFIREPSTETSTPPPPPPAQPTPQPQPHPPPQPETPPCAPSPPPPTPPCAPSPSPPTPPPGSPHKPEPQTPVYPEVPRS
TRSPPPSPPPTESAPEAPPSDTPSCRVPPCSSSPR**SGSQPKPPQDNTTTPK**MPSLSSPPTEHSTAQPPKHENDAR**EQEPPTDEDDFSSVK**GKKLGAYESDGSPRAS
SCAGAGVLGVFFMVVGLTV

## HSP70

MADSPAVGIDLGTTYSCVGVWK**NDAVEIIANDQGNRTTPSYVAFTDTERLVGDAAKNQVARNPENTIFDAKR**LIGR**KFDDPSVQSDMK**HWPFK**VIAGPGDKPLIEV**
**TYQGEKKTFHPEEVSAMVLGKMKEIAEAYLGKEVKEAVITVPAYFNDSQRQATKDAGTIAGLSVLRIINEPTAAAIAYGLDKK**GCGEMNVLIFDMGGGTFDVSLLT
IEDGIFEVK**ATAGDTHLGGEDFDNRLVDFCVQDFKR**KNRGKDISTNSRALRRLRTQCERTKRTLSSSTQATIEIDSLFEGIDYSVSISR**ARFEELCMDYFR**NSLLP
VEKVLKDSGIDKR**SVSEVVLVGGSTRIPKIQQLITDFFNGKEPCRSINPDEAVAYGAAVQAAILK**GVTSSQVQDLLLLDVAPLSLGLETAGGVMTKLIERNTTIPT
KK**SQTFTTYADNQPGVLIQVYEGERAMTK**DNNLLGKFHLDGIPPAPRGVPQIEVTFDIDANGIMNVTAQDKSTGK**SNQITITNDKGR**LSASEIDRMVQEAEKYKAE
DEQNKHRVEAK**NGLENYCYHMR**QTLDDEKLKDKISSEDRDTANKAIQEALDWLDKNQLAEKEEFEAK**QKEVESVCTPIITK**LYQAGAAAGGMPGGMGGMPGGMGGM
PGGMGGMPGGMGGMPGGMGGMPGAGMGGSGGPTVEEVD

## Chaperonin protein BiP

MTAAKKLSLFSLAALFCLLSVATLRPVAASDAEEGKVKDVVIGIDLGTTYSCVGVYR**HGRVDIIPNDQGNR**ITPSYVAFTDDDRKIGEAAKNEATINPTNTLFDVK
RLIGRRFNEKEVQKDKDLLPYEIINKDGKPYIRVMVKGEPKVLAPEEVSAMVLTKMKETAEQFLGKEVK**NAVVTVPAYFNDAQR**QATKDAGAIAGLNVIR**IINEPT**

**AAAIAYGLDKK**NEKTILVYDLGGGTFDVSVLVIDNGVFEVLATSGDTHLGGEDFDQRVMDHFIKLVKKKYDKDLRTDKRGLQKLRREVERAKRALSSQHQAKVEVE
NLMEGVDFSETLTR**AKFEELNSDLFQK**TLKPVKQVLEDADLQKSQVDEIVLVGGSTRIPKIQQLIKDFFNGKEPNRGINPDEAVAYGAAVQAGILSGEGAQDMVLL
DVTPLTLGIETAGGVMAKIINKNTVIPTKKTQTFSTYSDNQSAVLIQVYEGERPMTKHNHLLGKFELTGIPPAPRGVPQIEVTFDVDRNGILSVSAVDKGTGKSEK
ITITNDKGR**LTPEEIER**MISEAEKFAEEDKKVKERVDARNALEGYLHSMKTTVEDKDKLADKIEEDDKKTILDKVTEAQEWLNTNPDADAEETRDKLKDVEAVCNP
IISK**VYGQSGGPGAGGAAGGADDDDYGGHDEL**

## Hypothetical (TgME49_265170)

MTPSSPPSRRAPPRVQR**GEIAGETAGMSGSRPDLSAVAPAPPTR**LLASSSTPPGSPSLDAKGKR**MLASSPSSPAPATPSR**DRRVVFVYALLFLDVIIIGSTSAQHS
FNPASLIDKVLLTHASFPSAPSLEADAADLPFHRLLPHSVKRELGLACVCGWAALVVLGILTLLCSTSSPPVPLLASAYGRRTAASEGEDGGVRKAGSEKTTTADF
AEERGSRGKKSPDKEEGGKRASKETGRSRVEAGVAAAVETKFREYEQVVLSQYADATLTQVKMRSFFIALATLLVFFIINFILQINVQCHRTKHRLSR**MHALAAEV**
**AEHSEKLQDVRANPAPSTLHALDPASERFSSDSASLVK**IAENLPSLPGQSKETRQALSSTQGEAASPSPLLGPESKAEEANTGETVSETEAPLPVQPLPAGPHADE
RTEKGEDAEEPNVGNQKKAEKEGFASNHGTKSPPQDNPPLIETQSSSAPPPSFSFSRPSSLESDSDLRELGGGMERSEADDSAGSQEAPRSPSYVYYRDFWFWRQL
LASCLQDAELQLSVAWIAMHVAGTIFCMHVTRDVTESLKATVEAFRLL

## ACT1

MADEEVQALVVDNGSGNVKAGVAGDDAPR**AVFPSIVGKPKNPGIMVGMEEKDCYVGDEAQSKR**GILTLK**YPIEHGIVTNWDDMEKIWHHTFYNELRVAPEEHPVLL**
**TEAPLNPK**ANRERMTQIMFETFNVPAMYVAIQAVLSLYSSGR**TTGIVLDSGDGVSHTVPIYEGYALPHAIMRLDLAGRDLTEYMMKILHERGYGFTTSAEKEIVR**D
IKEKLCYIALDFDEEMKAAEDSSDIEK**SYELPDGNIITVGNERFRCPEALFQPSFLGKEAAGVHRTTFDSIMKCDVDIR**KDLYGNVVLSGGTTMYEGIGER**LTKEL**
**TSLAPSTMKIKVVAPPER**KYSVWIGGSILSSLSTFQQMWITK**EEYDESGPSIVHRK**CF

## GAPDH2

MSLYPRSSVRLRTRGLFFSSSLPLSHCLFLALFLFLSFDNFLPTSRSLNSFSVPAAALRLDGRDGPRPQTPETFSSSAFAEPSPESTPGACPLSSNDGELSLQQAE
ALLREEQTLATEYAQRAAGDVQQFTAAAQEAAARGDMSAAKELAAAAAASAAEAEDLAAYAQAVGETDPAVLRERAKEEAERFEQEERRQRGGDAASSGTCSESSD
AAFAPEFSDLRDLFFGACDATVSPDDLPSVPEALEKLAGLESDVQARGDQLDEKEKVVAEYLVAAQEAAVRHEEASRAFEDAKRNKKENLAAAAEKAALEAAAEYA
DLTTAAQLVTEEAEQESAKALADAVAAGDLRFAINQKIVNEAGPEARGKVPRALLAPKHVRISDPAAGRVLKIFFHNNFAPFEDLPCAALHADAHLCRQHPRCVVQ
VEQSGDEVLQRCLLDEAYLALLTKTPDFCAAAPAEAETGLGNSAAQATGTPVSPFAGAAPLEPMAAVTEGEIEAVALDLLQESGVDGPAVLEHMARDGRGSASDLC
SLVQTLATELLQTERDPRCAAAPTSEDRENIALTTEALLTSAFGFLGPASPSATAATAATVASGTPQNARSASIRSHRRNSFAPTGKRAVAPVPRVSPTGLFGLLG
SSSEKASAPIRLGINGMGRIGRLVFRIAMSRPDVAVTHINCSMDPAYIAYMLKYDSVHGKFDGEIVPTETSLIVNGQEVTVSNTR**DPEEIPWADK**GADYVCESTGV
FCTTEAAAKHVNRPGGAKHAIISAPAKDETTPTLVVGVNAEQDYESSMKVVSCASCTTNGLAPLVKVIDENFGLVEGLMTTVHAATGTQKVVDGTSKKDWRGGR**AA**
**AGNIIPSATGAAK**AVARCLPHMKGKLTGMAFRVPTLDVSVVDLTCRLNKSTTYEEIKKAVREASETYMRGIIGYTEEPIVSQDIVGSQCSTVFDANAGIMLNPNFV
KLVSWYDNEYAYSAR**LVDLIAVMAAKDGVVSPGTGLDR**RPF

## Hypothetical, MIC20 (TgME49_283540)

MHTKMTDGAKLYRRSMTTATTTGFFLLACWFLPSSCIQGSEALSTTATTQPISNVTRYPEQHTVAR**SAADVAKALAEK**VAR**EAMKNAYNDVFRNWTGIAHPDFHNG**
**DAETCKIVLSFDVAK**KK**NAMGSVTKILSVTVNNAR**GQLTVK**YLERPNVR**NATNVRRASSWVRRKLALETADEVVSVTETIDVPQACIADNHSR**YFVVTRR**K**NVIDT**
**RVFAVVIELELHPGPYLVPR**DISRIAELDRILK**EHERETFTEGKWVTLPGCMMLIR**VRETLKRAPR**LTIDAYRGSVQFREDELKSLGSQALPQGCTWDGPSAVGVV**
**TRHDRSADANLWDVLLFMADAPSHVQFEYTLPKEADVERNSDLSSGFASGPGEQDDNI**

## Hypothetical, IMC25 (TgME49_218240)

MRPAFSGTSQGAGTRALWRWGRRSSFVSLCAASFLGVILLGSAQESLSAPPQKAADSAASSQWPFGFLSGSGPTALLTSLAAPPTSDSGAPLAGRNDENRDPELGK
GDFLQPEESDNEGSAASPNRLPGPQQQELGEKGNAASRETAELSHSVTDSSPLFAVNARDAQSTLLSLWRKKKNEEAPLPSVASPISQAPAALQSALPFAAWFGTN
GDEDLHAGRSPSHANLVHSGNKAPSPATGEAAATASADRQSASWLPSFLSKKAQTDVGMQAFDSLLNSWFHSLRRLLGIPDGGDAALAAANAWESFQGDKSFWREL
SGDTRNASPLALARAAAWWFSQREREVENMARVAAGEGTQTVEHAAGVAAASAHAFWHRSGGAQASQETRAALEASAAWFRQQRNKLEQLDADGAAAWWESLTRSP
DNLPSSAKQQSAALGAAANVSSGVQQWFRGDEAQRVEGRKATGSMAFLTSESLGLGGPKMDKSRQSAWLSLWKGNEASNSPGDFAAAQVVSPEAAAAAAASWLDWS
RAREEETPSPPAPALSWFWSKQEQAVQEAVKNGALWLQKISEAPGAREVKNRAPAVRHSASSWVHSWLSSAGKGAKEVQEAEKQEGGEPDRESEGGVSFFSRWFGQ
KSSPIQEEKADGAAQDSLQRPEAASRWWLGQASSESNANKKEEALGSEAHEQVADNLLASTPNENEHSAGGDKAKEPEDTSGWFFQQKTFSDPNYPNKAGQAHQIA
EDVPQVKKGSAWLTDGEFVDGKAESSKETGRVDTSLLAQASQVLAQKAPQVSGWIFGGASEPEANGEDPAAHLVPGLKQGNSWWEWETSQTTTTTKTPAYTRDVK**L**
**DCNPVPPFR**ACVEKCQRDNRSREK**DKAGPSAPQALGSYEYGSLR**SCYLTCKQK**WIDTDLPGCLR**ADGVKVAGVPPVEAFRSTTSTTTSSTTTTTTHPPRTEPKAKE
PTAEAAK**SFWGGLSDKPSASEDLGDAPQEGIKSFFSIFQR**ATSTSTATAPPEAPEGFSLTKLLGFHRPETDTVSPGTASSNSFPFSLRGASTLWSARNDSKSEVGG
GLLGSLFRRNVSEVPKLAAANGVEEANVASAFSSTEDEEHASAGGSWWSSLSRGNDAEGRKDEKEAASGVARVPGKRSVEEAADAAAASAKDLVDEADDAVESVLS
TWWPVFLLVVGMGLLGLCVLGRRLGQWEGMAGVGDIERGEYASAPVFREGDSGKHEDNPRRSVDVLANDDNRRPLIEQSA

## ROP13

MKRTELCIAALVAVGAFAFTSPNAVAKSFERSLGHLDASSFLSSPLNSDVELGRSTFPAQSLSFTEGTNETNPPTSRPPGWKYEGSDLQRRVAARREEHKKRQEEW
EQRKASRRSALTPSAPDPDGDGDPATSFPSQRRLLDRCLQQFREQLVDLENLCKGSPEPDDCRSTVQEILGKQSFGALHTTVISFSIFVNRDPRR**LSFPVLDATDL**
**R**LTVKLKHLLDRIPGCAALSLPVYIGLVSSDVFKSEEFTRKVNRCSEDFGRSAREEPSRAGRAAAAVIRFMGLTPERQTFYQPFMFVTTQAAMLLSMVLKHPFLSI
LVNMACVAGSLCRKGIREVLLRALREADFLTEDVPLDSAPQELVDHLKVYLKLLFLRKYRRLRRQAANVAAQVVYANSLRLL
